# Effect of Mobilization With Movement on the Glenohumeral Joint Positional Fault in Subacromial Impingement

**DOI:** 10.7759/cureus.62576

**Published:** 2024-06-18

**Authors:** Shivanee Dalvi, Sandeep Shinde, Sumeeran D Mishra

**Affiliations:** 1 Department of Musculoskeletal Sciences, Krishna College of Physiotherapy, Krishna Vishwa Vidyapeeth (Deemed to be University), Karad, IND

**Keywords:** shoulder impingement, scapular stabilization exercises, chronic rotator cuff tear, acromion tuberosity index, acromion humeral distance

## Abstract

Introduction

Subacromial impingement (SAI) is a prevalent shoulder condition characterized by mechanical compression within the subacromial region. It presents with symptoms like shoulder pain and restricted motion, impacting a significant portion of the population. Neer's classification delineates three progressive stages of SAI, ranging from bursa edema to chronic rotator cuff tears. The etiology involves intrinsic and extrinsic factors, leading to altered kinematics and impingement. The study aims to determine the effect of mobilization with movement (MWM) on the glenohumeral joint positional fault in patients with SAI.

Materials and methods

The study comprised 80 participants diagnosed with SAI, selected based on the inclusion and exclusion criteria, and randomly divided into two groups, each consisting of 40 subjects. Group A received conventional therapy, while Group B received MWM in addition to conventional therapy. Treatment sessions, lasting 45 minutes, were administered five times weekly for four weeks. Pre- and post-treatment assessments included the visual analog scale (VAS), range of motion (ROM), acromion humeral distance (AHD), and acromion tuberosity index (ATI).

Results

The results demonstrated that there was an extremely significant improvement in VAS, shoulder ROM, and ADH in both groups, with a p-value of 0.0001, except for the ATI, which showed significant improvement in Group B with a p-value of 0.0001 compared to Group A.

Conclusions

Based on statistical analysis, the study found that MWM significantly improved joint positioning fault and has been beneficial in reducing pain and improving ROM.

## Introduction

Subacromial impingement (SAI) is defined as a mechanical compression injury of the tissues of the subacromial region [1]. It is the second most frequent shoulder condition, accounting for roughly 13.8% of cases. SAI presents with the symptoms of pain and restricted motion of the shoulder. According to the study done by Dutch practitioners and statistics from primary health care centers, 14.7 out of every 1000 individuals experience shoulder pain each year, with a lifetime incidence of up to 70%. Around 20% of adults in the community currently experience shoulder issues, and this number seems to be increasing [2]. Shoulder pain accounts for 44-65% of all complaints [3]. Any alteration in the subacromial space causes SAI. SAI includes a spectrum of subacromial space pathologies, including rotator cuff tendinosis, partial thickness rotator cuff tears, calcific tendinitis, and subacromial bursitis. Neer postulated three progressive types for this SAI they are as follows: subacromial bursa edema, which frequently occurs in people under the age of 25, is referred to as stage I of the process. Patients between the ages of 25 and 40 years old, who have stage II impingement, have permanent alterations that manifest as tendinitis and fibrosis of the rotator cuff tendon [4].

Stage III impingement is defined as persistent abnormalities, such as rotator cuff tears, which are typical in individuals older than 40 [4,5]. SAI has a complex etiology that can be classified as intrinsic or extrinsic and will then emerge as weakening, muscle imbalances, osteophytes, acromial changes, and altered kinematics leading to impingement [6,7]. The first, referred to as intrinsic impingement, suggests that partial or full-thickness tendon tears develop from a degenerative process brought on by repeated usage, tension overload, or trauma to the tendons over time. In contrast, extrinsic impingement occurs when the tendon becomes inflamed and degenerates due to mechanical compression by a structure outside of the tendon. Acromial or coracoacromial arch pathology, improper posture, changed scapular or glenohumeral kinematics, posterior capsular tightness, and other extrinsic mechanics are all possible causes of SAI [7,8].

Theoretically, an increase in normal superior and anterior humeral head translation would amplify these alterations in the subacromial region, resulting in mechanical compression of the tissues in the subacromial area during glenohumeral motion. Along with this, the scapular thoracic biomechanics are altered. Excessive superior and anterior humeral head translations would reduce the extent of the subacromial space, putting the subacromial tissues under further mechanical stress [9,10]. Altered kinematics in the humerus and scapula can result from several factors, including rotator cuff weakness, acromial morphology (flat type I, curved type II, or hooked type III), capsular tightness, shoulder instability, impaired scapulohumeral rhythm, scapular instability, and capsular-ligamentous laxity [11].

Six degrees of freedom, three rotations, and three translations are available in the glenohumeral joint. For physical functioning, the upper extremity is a crucial component of our body [12]. The upper extremity is used for a number of daily tasks, including writing, picking up objects, transferring objects from one location to another, eating, combing, bathing, and many other tasks [13]. In order to accomplish this work, individuals must repeatedly lift and carry objects overhead. To perform this work efficiently, complete shoulder range of motion (ROM) and strength of the rotator cuff muscles are important components [14]. Thus, SAI has a huge impact on socioeconomic status due to an individual's working ability limitation [15]. Various physical therapy interventions, which include thermotherapy, electrotherapy, therapeutic exercise therapy, and manual physical therapy, are used to treat impingement of the shoulder. Therapeutic exercises generally have a positive effect in restoring shoulder mobility and retraining muscle imbalance in impingement. Exercise is the first choice to improve function, pain, and ROM in the treatment of shoulder impingement. The reduction of pain can be enhanced in the short term by adding mobilizations with exercises [16]. Mobilization with movement (MWM) is a manual therapy technique used for musculoskeletal pain management. The therapist applies a sustained glide to a painful or stiff joint while the patient performs a concurrent active movement. The principles of MWM are based on analyzing and correcting any minor positional fault in the joint [17]. According to Mulligan, joint dysfunction or damage causes a positional fault or a chronic condition of misalignment within the joint [18]. The technique may help to realign the joint or restore its tracking mechanism MWM works on the positional fault theory. A positional fault is the micro malalignment of the two joint surfaces. Hence, it is important to have muscular balance around the joints. According to the literature, there are joint positional faults that occur at the glenohumeral joint along with scapular malalignment. To address the issue of altered shoulder kinematics, acromial humeral distance (AHD) and acromion tuberosity index (ATI) are used as investigative tools. A new radiographic measurement, ATI, was utilized to examine the association with rotator cuff pathology. ATI has proven to be an accurate and reliable predictor of degenerative supraspinatus tendon tears or SAI syndrome, making it a valuable tool for addressing these faults. This study aims to incorporate MWM and scapular stabilization exercises to improve shoulder pain, ROM, AHD, and ATI in individuals with SAI syndrome. The significance of this study lies in its potential to enhance diagnostic accuracy and therapeutic outcomes for patients suffering from SAI syndrome [19].

## Materials and methods

This study commenced after the approval from the Institutional Ethics Committee of Krishna Vishwa Vidyapeeth, Karad, India (Protocol No. 105/2022-2023). The study was carried out between August 2022 and April 2023 and included 80 participants who were selected based on inclusion and exclusion criteria. The inclusion criteria included males and females between 30 and 50 years of age. In diagnosed cases of SAI, ≥3 of 5 impingement tests were positive (Neer’s impingement test, Hawkins-Kennedy test, positive horizontal adduction test, Jobes test). The term painful arc describes pain experienced during shoulder abduction at an angle of 60° to 120° in subjects, with more than ≥2 planes of restriction. Subjects with painful and restricted internal or external rotation. Participants were excluded if they had limited shoulder ROM in two planes, a history of upper extremity fracture, shoulder surgery, full-thickness rotator cuff tear, shoulder instability, systemic musculoskeletal disease, shoulder pain associated with cervical spine motion, or scoliosis.

The individuals were randomly divided into two groups, namely Group A and Group B, using a simple random sampling method through IBM SPSS Statistics for Windows, Version 26 (Released 2019; IBM Corp., Armonk, NY, USA). This software was employed to ensure a random and unbiased allocation of participants to each group, enhancing the study's reliability. Additionally, a double-blinding method was implemented to minimize bias. Participants in Group A received conventional therapy. Participants in Group B received MWM along with conventional therapy. The treatment session was given for 45 minutes five times a week for four weeks. The pre and post-treatment assessments were done using the visual analog scale (VAS), ROM, AHD, and ATI. Post-assessment was conducted during the final session of the fourth week, and results were recorded. Follow-up with the patients took place 15 days after this last session.

Exercise protocol

Table 1 describes the exercise protocol for the conventional group, referred to as Group A [10].

**Table 1:** Exercise protocol for Group A reps: repetitions; secs: seconds

Week	Exercise	Repetition and hold
Week 1 (1-7 days)	Ultrasound for 6 minutes for 0.8 watts/cm^2^ for the anterior lateral aspect of the shoulder	6 minutes
	Cryotherapy for 10 minutes	10 minutes
	Crossed arm stretch	15 secs hold x 3 sets
	Shoulder shrug	10 reps x 3 sets
	Scapular retraction	10 reps x 3 sets
	Scaption	10 reps x 3 sets
	Wand exercise: Shoulder abduction in the scapular plane, shoulder flexion, shoulder external rotation, shoulder extension, and shoulder internal rotation (within the pain-free range)	10 reps
Week 2 (8-14 days)	Crossed arm stretch	15 secs hold x 3 sets
	Shoulder shrug	10 reps x 3 sets
	Scapular retraction	10 reps x 3 sets; 10 reps x 10 secs hold
	Scaption	10 reps x 3 sets (yellow resistance-band)
	Dynamic hug	10 reps x 3 sets (yellow resistance-band)
	Wand exercise: Shoulder abduction in the scapular plane, shoulder flexion, shoulder external rotation, shoulder extension, and shoulder internal rotation (within the pain-free range)	10 reps
Week 3 (15-21 days)	Crossed arm stretch (posterior capsular stretch)	30 secs hold x 3 sets
	Scapular retraction	15-15 reps (yellow resistance-band)
	Scapular retraction	10 reps x 10 secs hold (yellow resistance-band)
	Scaption	10 reps x 3 sets (red resistance-band)
	Dynamic hug	10 reps x 3 sets (red resistance-band) (standing)
	YTW exercises	10 reps x 3 sets
Week 4 (22-28 days)	Crossed arm stretch (posterior capsular stretch)	30 secs hold x 3 sets
	Scapular retraction	20-20 reps (red resistance-band) 10 reps x 10 secs hold (red resistance-band)
	Scaption	20 reps (red resistance-band)
	Dynamic hug	15 reps x 3 sets (red resistance-band)
	YTW exercises	10 reps x 3 sets (red resistance-band) (standing)

Statistical analysis

The sample size was calculated using the formula 4pq/L^2, and the collected data was entered into an Excel sheet (Microsoft® Corp., Redmond, WA, USA) and analyzed with SPSS software. Mean values and standard deviations were computed for each group. Data analysis utilized several techniques: a paired t-test was conducted to assess significant variations between pre-and post-intervention within the same group, while unpaired t-tests were used to identify significant differences between the groups. An independent sample t-test was used to compare outcome parameters before and after intervention, and the arithmetic mean and standard deviation were calculated for each outcome measure. A significance threshold of p < 0.05 was applied to all variables.

## Results

Table 2 presents the demographic data of the participants, showing the distribution of participants by age and gender. Specifically, there are 34 females and 46 males, with 43 participants aged between 30 and 45 years and 37 participants aged between 46 and 60 years.

**Table 2:** Demographic data of the participants

		Number of individuals
Age-wise distribution of participants	30-45 years	43
	46-60 years	37
Gender-wise distribution of participants	Female	34
	Male	46

Table 3 illustrates significant improvements in the VAS; both groups showed statistically significant reductions in pain levels both at rest and during activity. Group A’s VAS scores at rest decreased from 4.65 ± 0.95 to 2.08 ± 0.72 and during activity from 6.09 ± 0.58 to 2.50 ± 0.80. Group B’s VAS scores at rest decreased from 5.06 ± 0.66 to 2.36 ± 0.76 and during activity from 6.30 ± 0.48 to 2.87 ± 1.00.

**Table 3:** Comparison of pre and post-test mean scores of VAS (at rest and on activity) within Groups A and B

Group	VAS	Pre-test	Post-test	Mean difference	p-value
Group A	At rest	4.65 ± 0.95	2.08 ± 0.72	2.57	<0.0001
	On activity	6.09 ± 0.58	2.50 ± 0.80	2.7	<0.0001
Group B	At rest	5.06 ± 0.66	2.36 ± 0.76	3.59	<0.0001
	On activity	6.30 ± 0.48	2.87 ± 1.00	3.43	<0.0001

The results in Table 4 indicate significant improvements in ROM across all measured movements (flexion, abduction, internal rotation, and external rotation) for both Group A and Group B following the intervention, with notable gains in flexion and internal rotation.

**Table 4:** Comparison of pre- and post-test mean scores of range of motion (flexion, abduction, internal rotation, external rotation)

Range of motion	Group	Pre-test	Post-test	Mean difference	p-value
Flexion	Group A	92.77 ± 12.25	105.6 ± 12.31	-12.82	<0.0001
	Group B	95.87 ± 12.06	114.17 ± 12.06	-18.3	<0.0001
Abduction	Group A	80.27 ± 10.37	101.8 ± 9.96	-20.925	<0.0001
	Group B	82.1 ± 11.14	106.3 ± 10.60	-24.225	<0.0001
Internal rotation	Group A	63.22 ± 7.78	81.17 ± 6.710	-17.95	<0.0001
	Group B	55.15 ± 8.85	66.77 ± 5.89	-11.625	<0.0001
External rotation	Group A	58.67 ± 9.40	69.47 ± 8.46	-10.8	<0.0001
	Group B	54.27 ± 7.02	64.97 ± 6.61	-10.7	<0.0001

Table 5 demonstrates significant improvements in both AHD and ATI for Group A and Group B following the intervention, with Group A’s AHD increasing from 6.46 ± 0.58 to 9.05 ± 0.37, and ATI from 1.01 ± 0.22 to 1.12 ± 0.12, while Group B’s AHD increased from 6.39 ± 0.40 to 9.15 ± 0.44, and ATI from 1.02 ± 0.02 to 1.18 ± 0.06.

**Table 5:** Comparison of pre- and post-test mean scores of acromion-humeral distance (AHD) and acromion-tuberosity index (ATI)

	Group	Pre-test	Post-test	Mean difference	p-value
AHD	Group A	6.46 ± 0.58	9.05 ± 0.37	-2.59	<0.0001
	Group B	6.39 ± 0.40	9.15 ± 0.44	-2.75	<0.0001
ATI	Group A	1.01 ± 0.22	1.12 ± 0.12	-1.1	<0.042
	Group B	1.02 ± 0.02	1.18 ± 0.06	-0.15	<0.0001

Table 6 compares post-test values between Group A and Group B for the VAS, AHD, ATI, and ROM. Post-test comparisons between groups revealed significant differences in ATI, flexion, internal rotation, and external rotation, indicating better outcomes for Group B in flexion and ATI, and for Group A in internal and external rotation. There were no significant differences between the groups for VAS at rest, on activity, and AHD, while the difference in abduction was close to significance.

**Table 6:** Comparison of post-test values of VAS, AHD, ATI, and range of motion between Groups A and B VAS: visual analog scale; AHD: acromion-humeral distance; ATI: acromion-tuberosity index

		Post-test of Group A	Post-test of Group B	p-value
VAS	At rest	2.08 ± 0.72	2.36 ± 0.76	0.0964
	On activity	2.50 ± 0.80	2.87 ± 1.00	0.0777
AHD		9.052 ± 0.37	9.156 ± 0.441	0.2627
ATI		1.12 ± 0.12	1.180 ± 0.061	<0.0001
Flexion		105.6 ± 12.31	114.17 ± 12.06	0.002
Abduction		101.8 ± 9.96	106.32 ± 10.60	0.052
Internal rotation		81.17 ± 6.71	66.77 ± 5.89	<0.0001
External rotation		69.47 ± 8.46	64.97± 6.61	0.009

## Discussion

SAI is commonly reported in the general population and is one of the most common causes of disability at work and during daily activities. The primary factor leading to impingement syndrome has been the abnormal motion patterns of the shoulder. According to the literature, there are joint positional faults in the glenohumeral joint that cause increased superior translation and increased anterior translation of the humeral head during arm elevation. Along with this, scapular malalignment is evident, often caused by scapular muscle weakness and poor posture, resulting in pain and restricted shoulder movements. MWM and exercises have shown significant improvement in decreasing pain and improving ROM and posture. The fundamentals of MWM revolve around the examination and correction of even minor positional faults within the joint, whereas exercises play an important role in maintaining the scapular position and humeral head in place after the correction of the fault [1].

According to Singh et al., a cross-sectional study was carried out to report the prevalence of various disorders causing shoulder pain in patients reporting to a tertiary care hospital. The results stated that the age of the patients ranged between 23 and 69 years, and a predominance of males was observed in SAI. The commonest cause of pain by SAI was 13.8% in India. The age group of 30-45 included 43 subjects, while the age group of 46-60 included 37 subjects. Among the 80 subjects, 46 were male and 34 were female [2].

In a study done by Liu et al., ATI was measured with magnetic resonance imaging (MRI) and X-ray analysis on subjects, with rotator cuff pathology and acute rotator cuff tears. The lateral acromial angle (LAA), acromion type, acromion index (AI), and critical shoulder angle (CSA) were measured to assess their correlations with the ATI. The change in ATI after acromion surgery was evaluated in both groups. The results stated that ATI is a good predictor of degenerative supraspinatus tendon tears or SAI syndrome [18]. Thus, in our study, ATI was chosen as one of the outcome measures as it was found to be accurate and reliable with a sensitivity of 0.687 and a specificity of 0.697.

We have found significant results in pre- and post-assessments. Group B who received conventional therapy and MWM showed significant improvement in distance with p =< 0.0001. MWM, combined with therapeutic exercises, can help to correct humeral head positioning and scapular mechanics, which are critical for maintaining adequate subacromial space. Increased AHD and ATI suggest a reduction in the mechanical compression within the subacromial space. This can be attributed to improved scapulohumeral rhythm, where the scapula and humerus move in a more synchronized manner, reducing the likelihood of impingement. The interventions likely facilitated better scapular positioning and decreased anterior or superior translation of the humeral head, thereby increasing the subacromial space.

A correlation study conducted by Thamyongkti et al. on supraspinatus tear, ATI, and ATD was done on subjects with a history of shoulder pain for more than six months, with AP radiographs. The result of the study demonstrated statistically significant differences in ATI, ATD, and CSA between the patients with a supraspinatus tendon tear group and those without a supraspinatus tendon tear group [20]. Similarly, the current study depicted that, in pretreatment, the distance measured by ATI was reduced in SAI.

Significant results were observed in Group B, which received MWM therapy. In contrast, Group A, which underwent conventional therapy, did not demonstrate significant pre- to post-treatment changes. Specifically, Group B showed highly significant improvements from pre- to post-treatment.

Another study by Houglum and Peggy was conducted to find out the underlying causes and also to build a treatment regimen to resolve secondary SAI. This study concluded that the effective treatment of secondary SAI is the identification and correction of all causes that is the correction of posture, including scapular posture and muscles that control, stabilize, and move the scapula [21]. A cross-sectional study by Kim et al. used scapular sets exercise (SSE) on AHD and scapular muscle activity in patients with SAI. These findings stated that the SSE could be used to increase the AHD and activity of the serratus anterior, middle, and lower trapezius muscles in patients with SAI [22]. In our study, we also focused on the scapular muscles strengthening with a resistance band and scapular plane exercises, which help in maintaining the corrected joint positional fault and also realigning the scapula in place. There were significant results found in the pre- and post-values of AHD in both Groups A and B, with p ≤ 0.0001.

A study by Ajith and Shika used ultrasonography measurement on subjects with SAI, who were treated with MWM posterolateral glide for the shoulder for six sessions. Outcome measures included were measurement of the AHD [23]. In the current study, MWM was given with exercises in both groups. There was a significant outcome obtained in the pre- and post-test values of AHD with p = 0.0001. Similarly, a review by Hassan et al. on the effect of the Mulligan technique on SAI syndrome included a total of 11 randomized controlled trials (RCTs). They examined the effect of MWM combined with exercise with or without taping against rotator cuff strengthening, ROM exercises, isometric strengthening, shoulder joint mobilization, and sham techniques. The results showed that a combination of shoulder MWM and a supervised exercise program (ROM and functional limitations) has a better impact on pain than exercise alone [14]. The present study incorporated MWM, posture correction exercises, electrotherapy, and scapular stabilization exercises using resistance bands over four weeks. This intervention showed significant improvement in both Groups A and B, effectively reducing pain and enhancing AHD, as well as improving ROM and thereby reducing disability.

The study was carried out in a single-centered area and needs to be carried out in collaborative multi-centered areas. Due to financial constraints, MRI was not taken as an outcome measure, which could have given a better interpretation. The results of this study demonstrated that there was an extremely significant improvement seen in pain, ROM, and AHD for both groups, with p <= 0.0001. While ATI showed extreme significance in Group B as compared to Group A. Hence, incorporating a combination of MWM and conventional therapy demonstrated effectiveness in treating SAI.

## Conclusions

This study highlights the effectiveness of combining MWM and conventional therapy in treating SAI syndrome. The cohort in Group B, which received both MWM and conventional therapy, exhibited significantly superior outcomes in terms of pain alleviation, enhanced ROM, increased AHD, and improved ATI compared to Group A, which was administered conventional therapy alone. These results indicate that the integration of MWM with standard exercises effectively rectifies joint positional faults, optimizes scapular mechanics, and ensures sufficient subacromial space. Overall, incorporating MWM with conventional therapy presents a promising strategy for managing SAI syndrome, emphasizing the importance of addressing both the mechanical and functional aspects of shoulder impingement.
